# Supplementary material for: Time-domain proton-detected local-field NMR for molecular structure determination in complex lipid membranes
Source: Magn Reson (Gott). 2023 May 12;4(1):115–27. doi: 10.5194/mr-4-115-2023 (PMC10583295; doi:10.5194/mr-4-115-2023)
Supplement: The supplement related to this article is available online at: https://doi.org/10.5194/mr-4-115-2023-supplement. [file mr-4-115-supplement.pdf]

Supplement of Magn. Reson., 4, 115–127, 2023
https://doi.org/10.5194/mr-4-115-2023-supplement
© Author(s) 2023. CC BY 4.0 License.

Supplement of

Time-domain proton-detected local-field NMR for molecular structure determination in complex lipid membranes

Anika Wurl et al.

Correspondence to: Tiago Mendes Ferreira (tiago.ferreira@physik.uni-halle.de)

The copyright of individual parts of the supplement might differ from the article licence.

SAMPLE PREPARATION

All the lipids used in this work were acquired from AVANTI LIPIDS as dry powders. The purity of 1-palmitoyl-2-oleoyl-sn-glycero-3-phosphatidylethanolamine (POPE), 1,2-dimyristoyl-sn-glycero-3-phosphocholine (DMPC) and perdeuterated DMPC (DMPCd54) was higher than 99%. The brain lipid extract was a chloroform:methanol extract of porcine brain with a designated composition of 9.6 wt% of phosphatidylcholine (PC), 16.7 wt% of phosphatidylethanolamine (PE), 10.6 wt% of phosphatidylserine (PS), 2.8 wt% of phosphatidic acid, 1.6 wt% of phosphatidylinositol (PI) and of remaining undesignated lipids. The multi-lamellar vesicles of POPE and of the brain lipid extract were prepared by simply mixing the lipid powder with water (40 wt% of water) and repeatedly centrifuging and mixing the system using a thin metal rod until the sample was visibly homogeneous. DMPC/DMPCd54 samples were prepared by co-dissolving equal amounts (wt %) of DMPC and DMPCd54 in chloroform. The solvent was then evaporated under nitrogen flow, in a sonicator heatbath above the lipid melting temperatures. To remove any remaining solvent, the resulting lipid film was kept at reduced pressure over night. For hydration, the glass vial containing the lipid film was then placed in a desiccator with a volume of approximately 1 l together with a separate glass vial containing 3 ml of water. The desiccator was evacuated and the lipid film kept in the resulting high humidity environment for at least six hours. The hydrated films were then filled into Bruker MAS sample inserts of 25 μl volume.

NMR EXPERIMENTS

The experiments were conducted on a Bruker Avance III spectrometer operating at a ^{1}H Larmor frequency of 400.03 MHz. The ^{2}H NMR experiments and the ^{1}H-^{13}C R-PDLF NMR experiments were performed by using a broadband static probe and a standard double channel 4 mm MAS solid-state NMR probe, respectively. All R-PDLF experiments were done on samples kept inside 25 μl Kel-F disposable high-resolution inserts purchased from BRUKER and using magic angle spinning (MAS) at a frequency of 5 kHz.

RF pulse callibration

RF pulses were calibrated by performing 2D NMR experiments to measure the nutation frequency at fixed RF power levels. The 2D NMR experiments used consisted simply on using a single pulse with increments of the pulse length in the indirect dimension. Fourier transformation in both the direct and indirect dimensions yielded splittings from which the nutation frequency was determined and used to calibrate all RF pulses. The nutation frequency of the resulting power levels for $R18_1^7$ pulses was verified immediately before and after the R-PDLF experiments were performed.

R-PDLF experiments

The R-PDLF experiments were performed using $R18_1^7$ recoupling blocks and rINEPT as the polarization transfer step [1]. The nutation frequencies of the $R18_1^7$ pulses was 45 kHz. The delays used for the rINEPT block were 1.2 and 2 ms and the rINEPT pulses had a nutation frequency of 78.125 kHz. To optimize the rINEPT step, it is crucial to use multiples of the MAS period for these delays. During acquisition heteronuclear decoupling was performed with SPINAL64 [2]. Depending on the sample, a total number of 8 to 32 points in the indirect dimension were measured with increments of twice the rotation period. For processing the data, the Fourier transform in the direct dimension was done after performing zero filling and apodization (with a simple single-exponential decay) of the free induction decays. The Fourier transform in the indirect dimension was processed after zero filling but no apodization was used.

^{2}H NMR experiments

For acquiring the ^{2}H NMR spectrum of the DMPC/DMPCd54 sample, we employed a quadrupole echo sequence [3] with a pulse nutation frequency of 58 kHz, an inter-pulse delay of 40 μs and a relaxation delay of 1 s. Signal acquisition was started directly after the second pulse, for a total of 50 ms with dwell time of 0.5 μs. For processing the data, we used the maximum of the quadrupole echo acquired as the first point of the time domain signal and discarded the previous points. The ^{2}NMR spectrum was obtained then by performing a Fourier transform on the time domain signal without using neither zero-filling nor apodization.

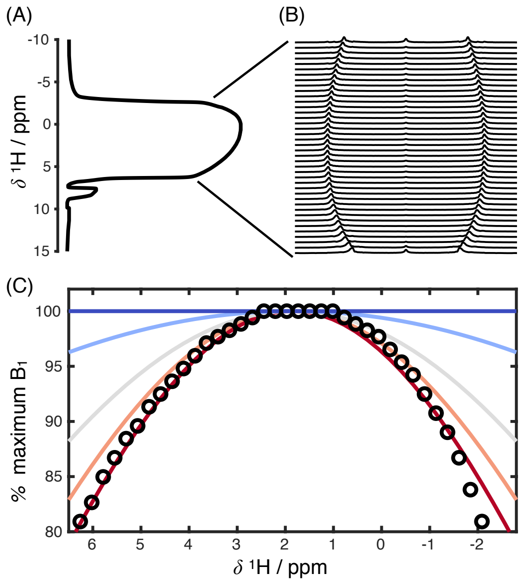

FIG. S1: Measurement of the RF field strength spatial inhomogeneity using a water sample contained in an insert. (A) Imaging of the sample by measuring the ^{1}H NMR spectrum of the sample under a magnetic field z-gradient generated with the z-linear shim coil. (B) Indirect dimension spectra showing the nutation frequency accross the different positions in the sample. (C) Experimental RF inhomogeneity profile (circles) and gaussian functions with different width (solid lines). The red line corresponds to the gaussian function used to describe the spatial inhomogeneity in the NMR simulations performed.

Measurement of the RF spatial inhomogeneity profile

To determine the RF spatial inhomogeneity of the double channel 4 mm MAS solid-state NMR probe, we used an insert for 4 mm solid-state NMR rotors (purchased from BRUKER) filled with water. The same type of inserts were used for all measurements such that all the samples investigated experienced the same exact RF spatial inhomogeneity. The RF field strength inhomogeneity was measured by applying the method proposed by Odedra and Wimperis [4], namely by using a strong linear magnetic field gradient along the direction of the external magnetic field (by means of the corresponding shimming coil) enabling 1D imaging of the sample and performing the 2D NMR pulse sequence described in the previous section for recording a spatially resolved nutation frequency profile. Fig. S1 shows the RF inhomogeneity profile measured which was fitted with a gaussian lineshape. Such gaussian profile was then used to generate R-PDLF simulation data that was representative of the experimental conditions (details in the next section). Similarly to previous studies [5, 6], the RF profile measured shows a plateau for the RF strength in the central part of the sample (the region of the sample with the highest nutation frequency), and a nearly symmetrical decrease of RF field strength towards the outer parts of the sample. By summing over the Fourier transforms of the spatially resolved slices one observes that the resulting splitting is equal to the maximum splitting i.e. the splitting corresponding to the plateau region. This means that if one performs the same 2D nutation experiment using typical shimming settings that enable high resolution (i.e. narrow ^{1}H peaks), the splitting observed in the indirect dimension yields the nutation frequency corresponding to the middle part of the sample where the highest nutation frequency is observed. We use this nutation frequency value to calibrate our RF pulses. Note that by simply finding the pulse length for which the magnetization crosses zero after the first maximum yields a lower nutation frequency leading to miscalibration of the RF pulses.

NMR SIMULATIONS

The NMR simulations of the R-PDLF experiments were generated with SIMPSON [7]. To account for the RF inhomogeneity in the simulations a MATLAB script was composed that generates SIMPSON input files and SIMPSON call-outs to cover the measured RF inhomogeneity profile of the used double channel 4 mm MAS solid-state NMR probe. Both the dipolar recoupling and the rINEPT blocks were included in the NMR simulations performed. The procedure generated a simulation database of R-PDLF simulations with inclusion of RF inhomogeneity for several dipolar couplings ranging from 0 to 22 kHz in steps of 22 Hz which corresponds to C–H bond order parameter steps of 0.001. For each distinct dipolar coupling value, a total number of 40 simulations were used to cover the RF inhomogeneity profile. Each simulation was performed with the following parameters.

The total number of time domain points simulated (indirect dimension) was 128. The number of repulsion angles used was 320 and the number of gamma angles was 6. All the remaining settings were the same as in the experiments performed. The simulation database generated was then used to fit the experimental data. The MATLAB script used and the database generated are accessible for public use in https://github.com/tfmFerreira/inhomogeneous-rf-nmr.git.

TIME-DOMAIN FITTING AND DEFINITION OF ERROR LIMITS

One component fits. In order to fit an experimental R-PDLF dipolar modulation assuming a one-component fit, we determined χ^2 defined as

$$\chi^2 \equiv \sum_{i=1}^{N} \left(\frac{y_i - y_i^{\text{sim}}(t_i; S_{\text{CH}}, R)}{\sigma} \right)^2 \tag{1}$$

where y_i are the experimental values measured for each time point t_i during the indirect dimension for a fixed chemical shift value, σ is the standard deviation calculated from the noise distribution inside a region of the spectrum with no peaks, and $y_i^{\text{sim}}(t_i; S_{\text{CH}}, R)$ is the R-PDLF simulated model for a C–H spin pair with order parameter S_{CH} multiplied by a phenomenological relaxation decay, $\exp(-2\pi R t_i)$. The fitted order parameter value was obtained by finding the minimum of χ^2 in the ranges $0 \leq S_{\text{CH}} \leq 0.5$ and $0 \leq R \leq 100$ Hz. The errors determined for the order parameters fitted were obtained by the following procedure. First, for each order parameter value, the minimum of χ^2 was determined by varying R. The set of values calculated define an uni-dimensional function χ_1^2 that depends exclusively on S_{CH} (note that negative and positive order parameters give the same result so $|S_{\text{CH}}|$ is the relevant quantity). The upper and lower errors for the fitted value were then defined from the order parameters at which χ_1^2 is equal to 2.71 which gives confidence levels close to 90% (see e.g. Chapter 15, section 15.6, page 692 in reference [8]).

Two component fits. In order to fit an experimental R-PDLF dipolar modulation assuming a two-component fit, we used the same procedure as above with the exception that $y_i^{\text{sim}}(t_i; S_{\text{CH}}, R)$ is a model corresponding to the sum of two R-PDLF simulated models for a C–H spin pair with distinct order parameters S_{CH}^a and S_{CH}^b multiplied by a phenomenological relaxation decay, $\exp(-2\pi R t_i)$. R was assumed to be the same for the two components. The fitted order parameter value was obtained by finding the minimum of χ^2 in the ranges $0 \leq S_{\text{CH}} \leq 0.5$ (for both components) and $0 \leq R \leq 100$ Hz. Similarly to the one-component case above, the errors determined for the order parameters fitted were obtained by the following procedure. First, for each set of two distinct order parameter values, the minimum of χ^2 was determined by varying R. The set of values calculated define a two-dimensional surface χ_2^2 that depends on S_{CH}^a and S_{CH}^b. The upper and lower errors for the fitted values for S_{CH}^a and S_{CH}^b were then defined from the boundaries at which the χ_2^2 surface is equal to 4.61 corresponding to confidence levels close to 90% (see e.g. Chapter 15, section 15.6, page 692 in reference [8]).

[1] S. V. Dvinskikh, H. Zimmermann, A. Maliniak, and D. Sandstrom, J. Magn. Reson. **168**, 194 (2004).
[2] B. Fung, A. Khitrin, and K. Ermolaev, J. Magn. Reson. **142**, 97 (2000), URL https://www.sciencedirect.com/science/article/pii/S1090780799918967.
[3] J. Davis, Biochimica et Biophysica Acta (BBA) - Reviews on Biomembranes **737**, 117 (1983), ISSN 0304-4157, URL https://www.sciencedirect.com/science/article/pii/0304415783900151.
[4] S. Odedra and S. Wimperis, J. Mag. Reson. **231**, 95 (2013), URL http://www.sciencedirect.com/science/article/pii/S1090780713000955.
[5] Z. Tošner, A. Purea, J. O. Struppe, S. Wegner, F. Engelke, S. J. Glaser, and B. Reif, Journal of Magnetic Resonance **284**, 20 (2017), URL https://www.sciencedirect.com/science/article/pii/S1090780717302276.
[6] K. Nishimura, R. Fu, and T. A. Cross, J. Mag. Reson. **152**, 227 (2001), URL https://www.sciencedirect.com/science/article/pii/S1090780701924103.
[7] M. Bak, J. Rasmussen, and N. Nielsen, J. Magn. Reson. **147**, 296 (2000), URL https://www.sciencedirect.com/science/article/pii/S1090780700921797.
[8] W. Press, B. Flannery, S. Teukolsky, and W. Vetterling, *Numerical Recipes* (Cambridge University Press, Cambridge, USA, 1992).

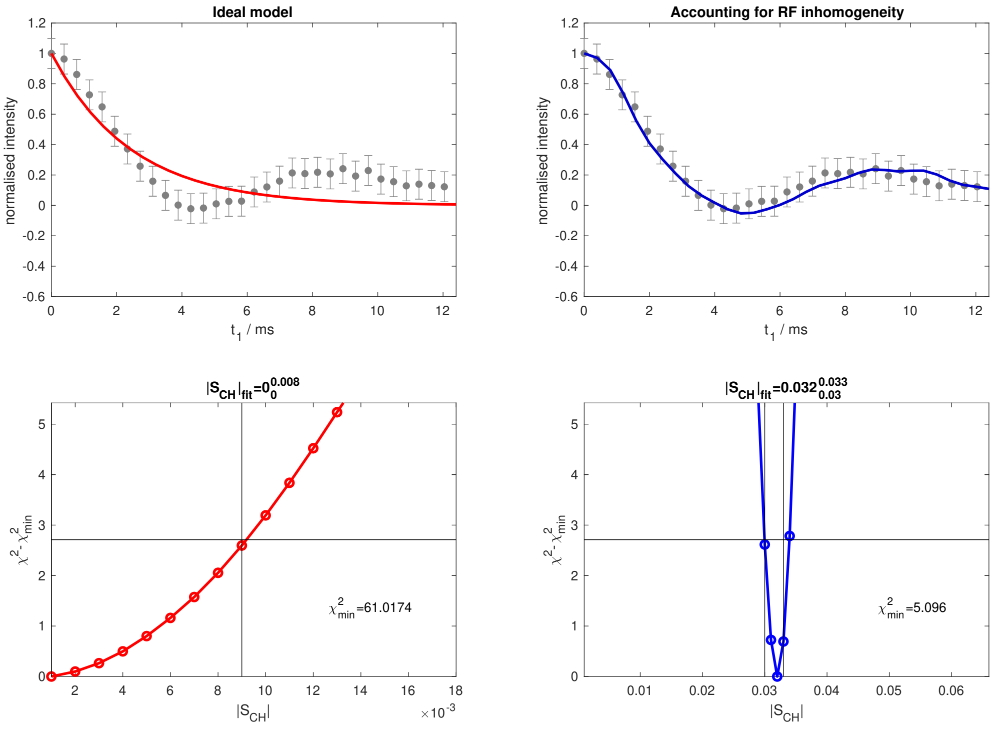

FIG. S2: One-component time-domain fits of the dipolar modulation of carbon β in the POPE sample and $\Delta\chi_1^2$ using an ideal model of the R-PDLF experiment (left) and a model that accounts for the RF inhomogeneity of the probe used (right). The superscript and subscript on the top of the bottom plots denote the limiting values obtained for $\Delta\chi_1^2$ equal to 2.71 (i.e 90% confidence bounds [8]).

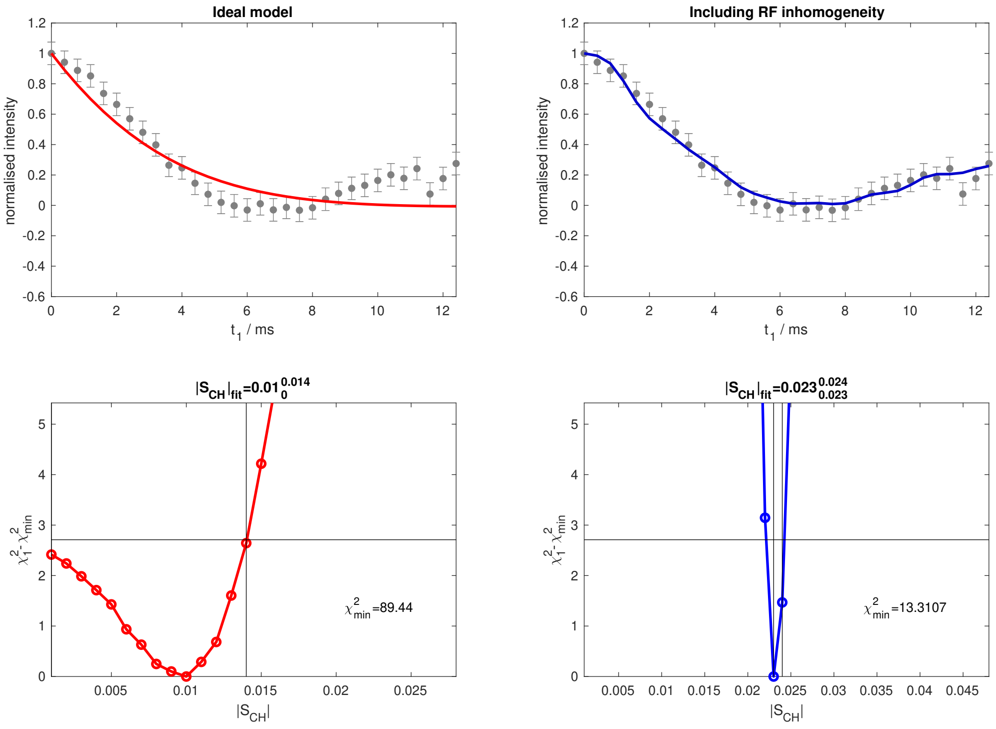

FIG. S3: One-component time-domain fits of the dipolar modulation of carbon ω in the DMPC/DMPCd54 sample and $\Delta\chi_1^2$ using an ideal model of the R-PDLF experiment (left) and a model that accounts for the RF inhomogeneity of the probe used (right). The superscript and subscript on the top of the bottom plots denote the limiting values obtained for $\Delta\chi_1^2$ equal to 2.71 (i.e 90% confidence bounds [8]).

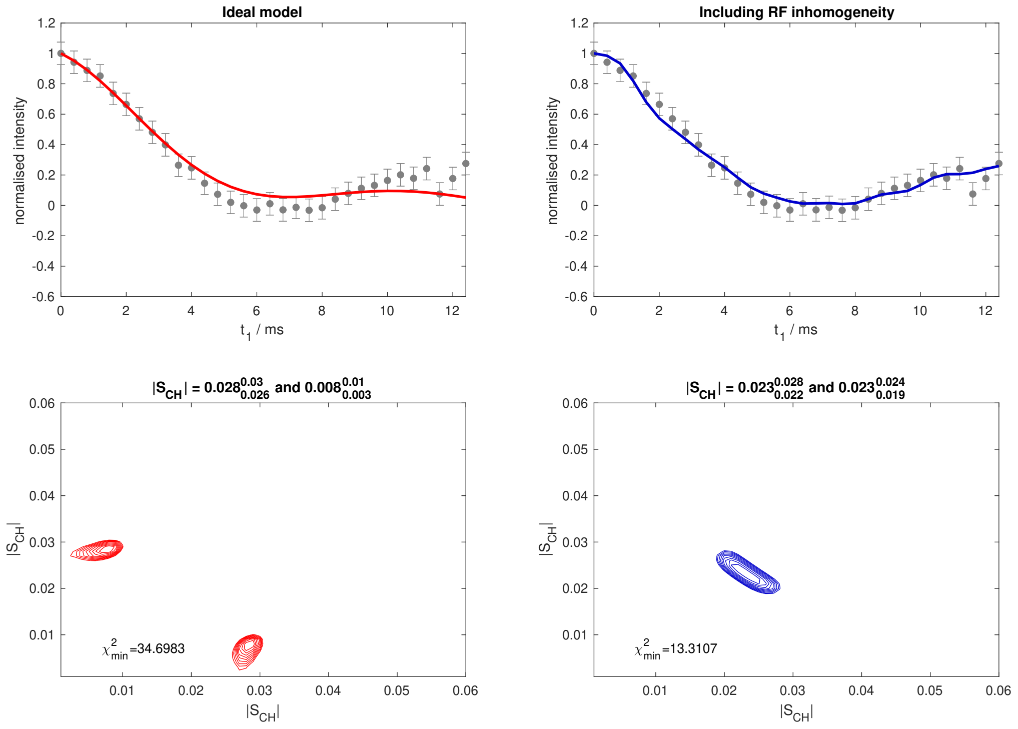

FIG. S4: Two-component time-domain fits of the dipolar modulation of carbon ω in the DMPC/DMPCd54 sample (top) and $\Delta\chi^2_2$ (bottom) using an ideal model of the R-PDLF experiment (left) and a model that accounts for the RF inhomogeneity of the probe used (right). The superscript and subscript on the top of the bottom plots denote the limiting values obtained for $\Delta\chi^2_2$ equal to 4.61 (i.e 90% confidence bounds [8]).

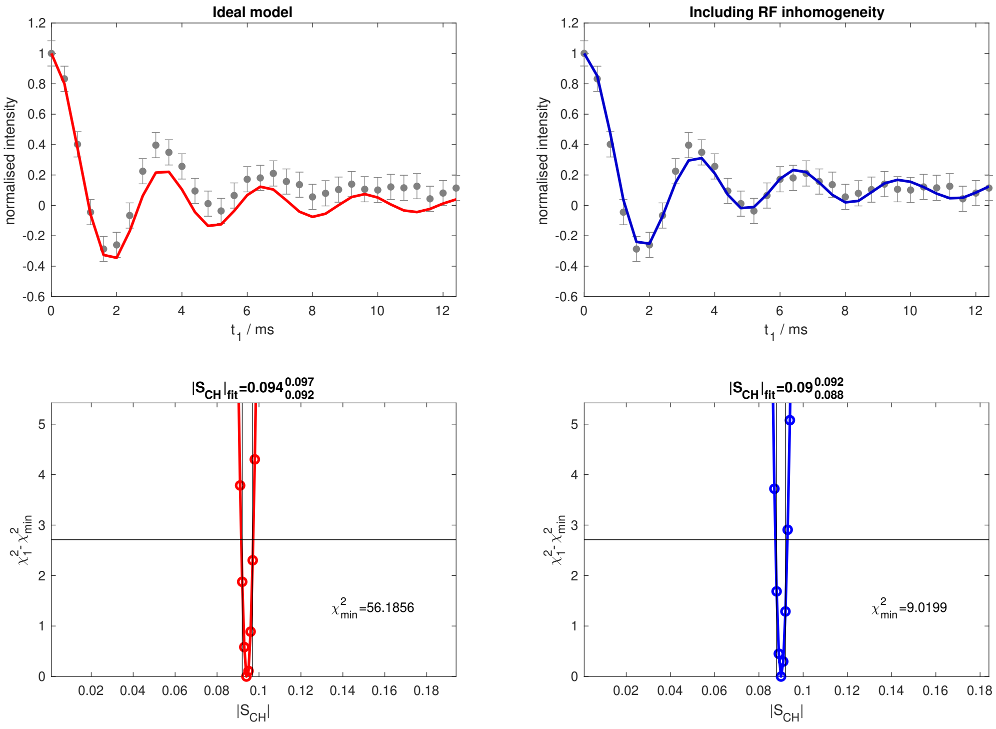

FIG. S5: One-component time-domain fits of the dipolar modulation of carbon $\omega - 1$ in the DMPC/DMPCd54 sample and $\Delta\chi_1^2$ using an ideal model of the R-PDLF experiment (left) and a model that accounts for the RF inhomogeneity of the probe used (right). The superscript and subscript on the top of the bottom plots denote the limiting values obtained for $\Delta\chi_1^2$ equal to 2.71 (i.e 90% confidence bounds [8]).

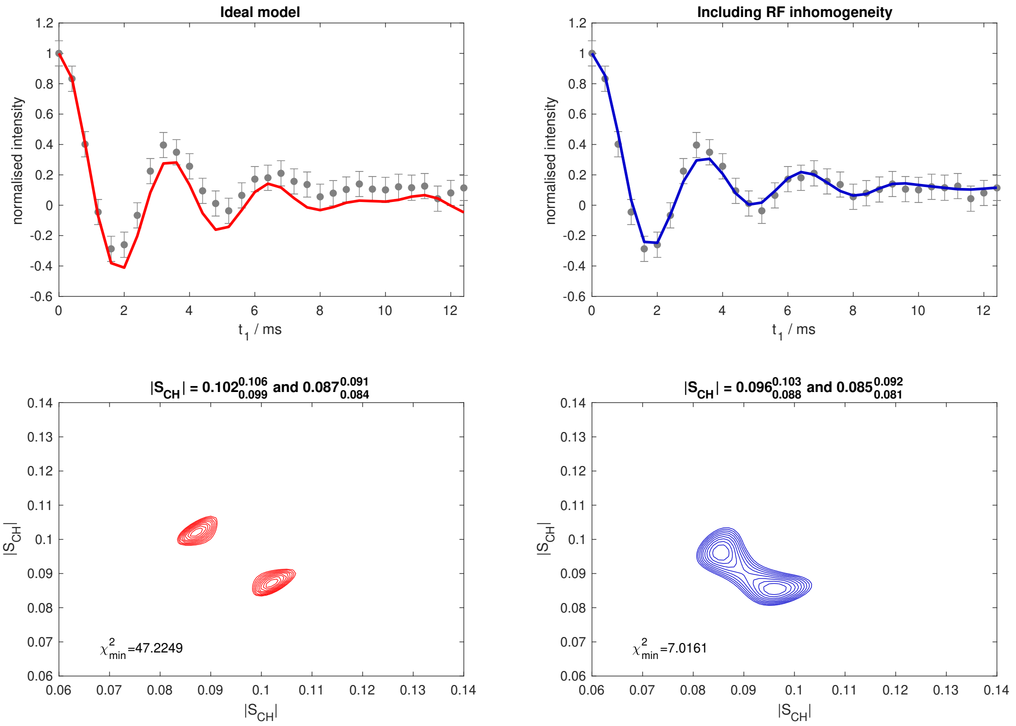

FIG. S6: Two-component time-domain fits of the dipolar modulation of carbon $\omega - 1$ in the DMPC/DMPCd54 sample (top) and $\Delta\chi^2_2$ (bottom) using an ideal model of the R-PDLF experiment (left) and a model that accounts for the RF inhomogeneity of the probe used (right). The superscript and subscript on the top of the bottom plots denote the limiting values obtained for $\Delta\chi^2_2$ equal to 4.61 (i.e 90% confidence bounds [8]).

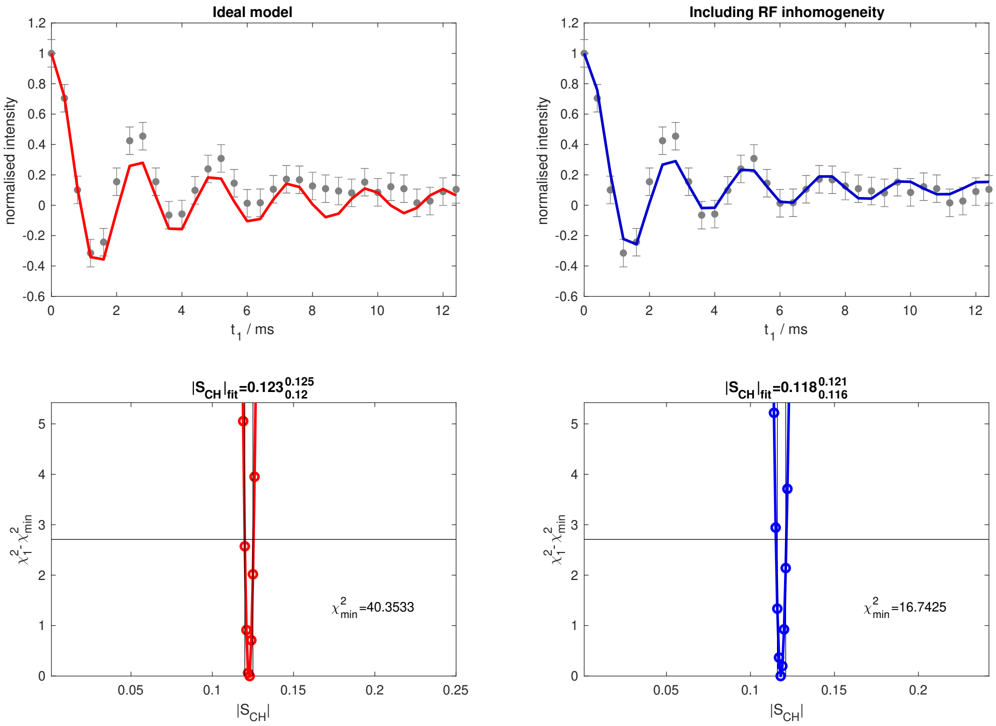

FIG. S7: One-component time-domain fits of the dipolar modulation of carbon $\omega - 2$ in the DMPC/DMPCd54 sample and $\Delta\chi^2_1$ using an ideal model of the R-PDLF experiment (left) and a model that accounts for the RF inhomogeneity of the probe used (right). The superscript and subscript on the top of the bottom plots denote the limiting values obtained for $\Delta\chi^2_1$ equal to 2.71 (i.e 90% confidence bounds [8]).

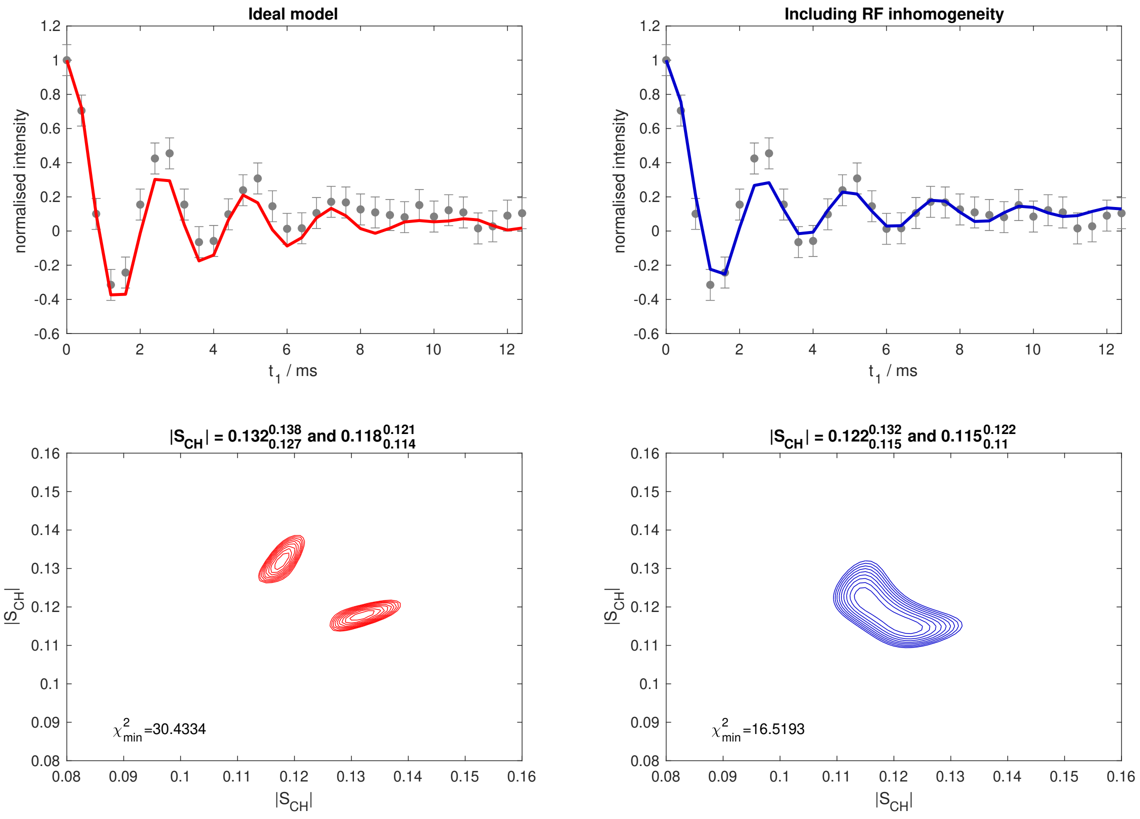

FIG. S8: Two-component time-domain fits of the dipolar modulation of carbon $\omega - 2$ in the DMPC/DMPCd54 sample (top) and $\Delta\chi^2_2$ using an ideal model of the R-PDLF experiment (left) and a model that accounts for the RF inhomogeneity of the probe used (right). The superscript and subscript on the top of the bottom plots denote the limiting values obtained for $\Delta\chi^2_2$ equal to 4.61 (i.e 90% confidence bounds [8]).

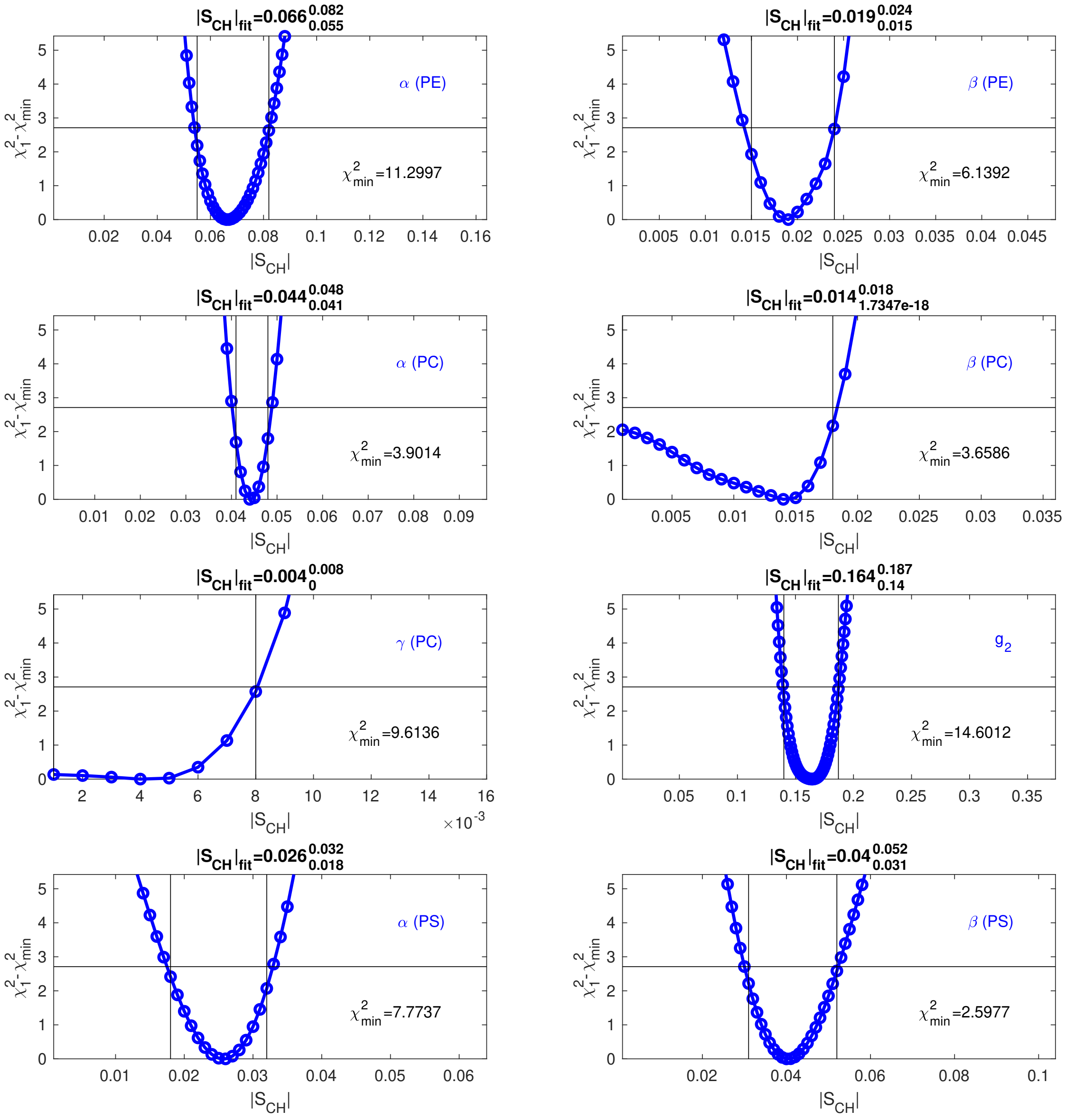

FIG. S9: Dependence of $\Delta\chi^2$ for the fits of the R-PDLF dipolar modulations shown in the main text for the sample composed of a brain lipid extract. The superscript and subscript on the top of each plot denote the limiting values obtained for $\Delta\chi^2$ equal to 2.71 (i.e 90% confidence bounds [8]).
